# The mitochondrial genome of a wild toxic mushroom, *Russula subnigricans*

**DOI:** 10.1080/23802359.2019.1692719

**Published:** 2019-11-20

**Authors:** Fei Yu, Yongjie Zhang, Junfeng Liang

**Affiliations:** a Key Laboratory of State Forestry Administration on Tropical Forestry Research, Research Institute of Tropical Forestry, Chinese Academy of Forestry, Guangzhou, China;; b College of Life Science, Shanxi University, Taiyuan, China

**Keywords:** *Russula subnigricans*, mitochondrial genome, phylogeny

## Abstract

*Russula subnigricans* is a wild toxic mushroom in the world. In this study, we present the 60,949 bp mitochondrial genome of *R. subnigricans* with a GC content of 21%. The mtDNA assembly consists of 60 genes, which including 14 standard protein-coding genes, two rRNA genes, 25 tRNA genes and 19 free-standing open reading frames (ORFs). Phylogenetic analysis was performed using 14 protein-coding genes, the results showed that the *R. subnigricans* had high homology with other *Russula* species.

*Russula subnigricans* is one of the most toxic wild mushrooms in the world, which can cause rhabdomyolysis (Lin et al. 2015). Some cytotoxic substances such as Russuphelin A, B, C, D, E, F were isolated from *R. subnigricans*, which Russuphelin were cytotoxic to various solid tumor cells (Takahashi et al. 1992, 1993). Matsuura et al. (2009) isolated cycloprop-2-ene carboxylic acid from *R. subnigricans* and demonstrated that the compound can cause fatal rhabdomyolysis. The mitogenome has been utilized for phylogenetic studies (Carpi et al. 2016), but no mitogenome information of *Russula subnigricans* was reported.

*Russula subnigricans* (strain CX24) was collected from Changxing County, Huzhou City, Zhejiang Province, China (119°53′E, 31°6′N). It was immediately frozen with liquid nitrogen and stored in the −80 °C refrigerator. Specimens were deposited in the Research Institute of Tropical Forestry, Chinese Academy of Forestry (RITF4153). Genomic DNA was extracted by CTAB method and its concentration was measured, and then sequenced using Illumina HiSeq x-10 of Shanghai Majorbio Bio-pharm Biotechnology Co., Ltd, China.

The mtDNA genome of *R. subnigricans* was assembled by MITObim V1.9.1 and MIRA V4.0.2, and *Russula compacta* (MH138072) was used as the reference genome. MFannot V1.33 (Valach et al. 2014) and MITOS2 (Bernt et al. 2013) were annotated mitogenome, and then NCBI was correct the coding protein gene and open reading frame. tRNAscan-SE2.0 was used to predict tRNA genes. Using mega v6.06, 14 protein coding genes (*atp6*, *atp8*, *atp9*, *cob*, *cox1*, *cox2*, *cox3*, *nad1*, *nad2*, *nad3*, *nad4*, *nad4L*, *nad5,* and *nad6*) were aligned (Kumar et al. 2008). With SequenceMatrix v1.8 concatenated polygene fragments, MEGA v6.06 constructed a neighbor-joining phylogenetic tree by 1000 bootstrap replicates based on polygene fragments.

The mitogenome of *R. subnigricans* was assembled into a circular molecule of 60,949-bp with a GC content of 21% (GenBank accession: MN565028). The base composition of mitogenome was 38.51% for A, 10.18% for C, 10.90% for G, and 40.41% for T. There are 60 genes, including 14 protein coding genes, two rRNA genes, 25 tRNA genes and 19 open reading frame (ORFs). The start codon of 14 protein coding gene was ATG, while the termination codon (TAA and TAG) were identified in 14 protein coding genes; TAG for *atp6* and TAA as stop codon for remain protein coding gene. The *R. subnigricans* mitogenome contained two tRNA genes with the common anticodons for methionine, two tRNA genes with different anticodons for leucine and serine, and three tRNAs with different anticodons for arginine. The mitochondrial genome of *R. subnigricans* contains five introns, two of which are in *cox1* and the remaining in *nad1*, *nad5*, and *cob*, respectively.

To validate the determined sequences, we conducted a neighbor-joining phylogenetic analysis based on 14 protein-coding genes. 20 closely species mitogenomes were chosen for and rooted with *Cantharellus cibarius* (Figure 1). *Russula subnigricans* clustered with other *Russula* species. These species grouped together with *Heterobasidion irregular* to form the clade of Russulales.

**Figure 1.**
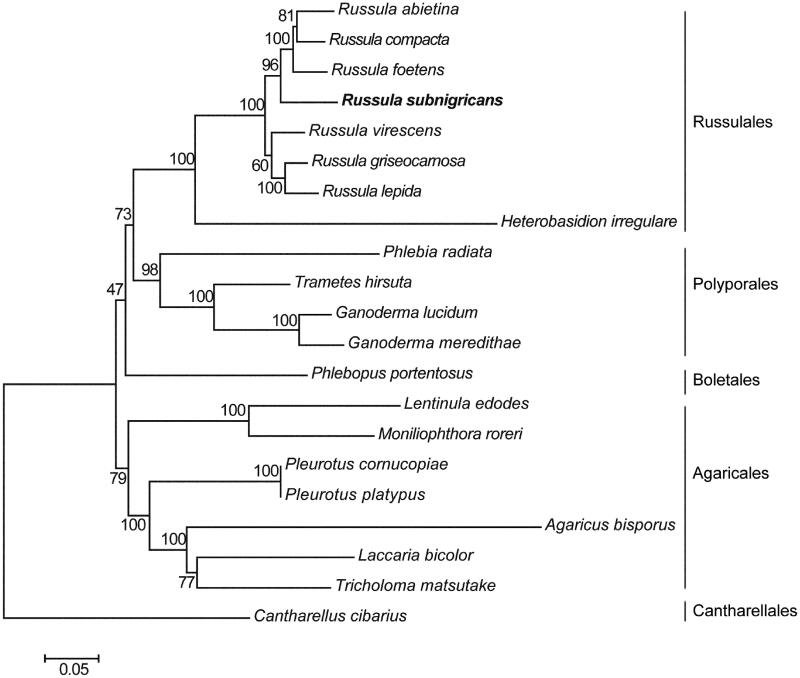
Phylogenetic analyses of 21 fungi based on 14 protein coding genes. GenBank accessionn numbers: *Agaricus bisporus* (JX271275), *Cantharellus cibarius* (KC573037), *Ganoderma lucidum* (NC_021750), *Ganoderma meredithae* (NC_026782), *Heterobasidion irregulare* (KF957635), *Laccaria bicolor* (NC_042773), *Lentinula edodes* (NC_018365), *Moniliophthora roreri* (HQ259115), *Phlebia radiata* (HE613568), *Phlebopus portentosus* (MK571437), *Pleurotus cornucopiae* (NC_038091), *Pleurotus platypus* (NC_036999), *Russula abietina* (MH138073), *Russula compacta* (MH138072), *Russula foetens* (MH138074), *Russula lepida* (MH138075), *Russula griseocarnosa* (MN427435), *Russula virescens* (MH138076), *Trametes hirsuta* (MG775432), and *Tricholoma matsutake* (NC_028135).
